# Chemical modification by peroxynitrite enhances TLR4 activation of the grass pollen allergen Phl p 5

**DOI:** 10.3389/falgy.2023.1066392

**Published:** 2023-02-15

**Authors:** Kathrin Reinmuth-Selzle, Iris Bellinghausen, Anna Lena Leifke, Anna T. Backes, Nadine Bothen, Kira Ziegler, Michael G. Weller, Joachim Saloga, Detlef Schuppan, Kurt Lucas, Ulrich Pöschl, Janine Fröhlich-Nowoisky

**Affiliations:** ^1^ Multiphase Chemistry Department, Max Planck Institute for Chemistry, Mainz, Germany; ^2^ Department of Dermatology, University Medical Center of the Johannes Gutenberg University, Mainz, Germany; ^3^ Federal Institute for Materials Research and Testing (BAM), Berlin, Germany; ^4^ Institute of Translational Immunology, University Medical Center of the Johannes Gutenberg University, Mainz, Germany; ^5^ Division of Gastroenterology, Beth Israel Deaconess Medical Center, Harvard Medical School, MA, USA

**Keywords:** allergy, Bet v 1, Phl p 5, nitration, dimers, oligomers, peroxynitrite, air pollution

## Abstract

The chemical modification of aeroallergens by reactive oxygen and nitrogen species (ROS/RNS) may contribute to the growing prevalence of respiratory allergies in industrialized countries. Post-translational modifications can alter the immunological properties of proteins, but the underlying mechanisms and effects are not well understood. In this study, we investigate the Toll-like receptor 4 (TLR4) activation of the major birch and grass pollen allergens Bet v 1 and Phl p 5, and how the physiological oxidant peroxynitrite (ONOO^–^) changes the TLR4 activation through protein nitration and the formation of protein dimers and higher oligomers. Of the two allergens, Bet v 1 exhibited no TLR4 activation, but we found TLR4 activation of Phl p 5, which increased after modification with ONOO^–^ and may play a role in the sensitization against this grass pollen allergen. We attribute the TLR4 activation mainly to the two-domain structure of Phl p 5 which may promote TLR4 dimerization and activation. The enhanced TLR4 signaling of the modified allergen indicates that the ONOO^–^-induced modifications affect relevant protein-receptor interactions. This may lead to increased sensitization to the grass pollen allergen and thus contribute to the increasing prevalence of allergies in the Anthropocene, the present era of globally pervasive anthropogenic influence on the environment.

## 1. Introduction

The prevalence and severity of allergic diseases triggered by airborne plant pollen and other allergens are increasing worldwide (1–5). Among others, a possible driving factor for this trend is the exposure of allergens to reactive oxygen and nitrogen species (ROS/RNS) caused by air pollution (5–8). Anthropogenic air pollutants like ozone (O_3_), nitrogen dioxide (NO_2_), and particulate matter can trigger or enhance oxidative stress and inflammatory processes that lead to the formation of endogenous ROS/RNS such as peroxynitrite (ONOO^–^) (5, 9). The ROS/RNS react with oxidation-sensitive amino acids of proteins, especially tyrosine, forming nitrotyrosine as well as intramolecular and intermolecular dityrosine cross-links, both known as markers of inflammation and oxidative stress (9–15). Besides modification by ONOO^–^, the tyrosine residues of the proteins can also be modified directly in the environment. For example, air pollutants can damage the pollen cell wall and facilitate the release of allergenic proteins and other cytoplasmic substances into the environment (16–20). The allergenic proteins can thus be directly exposed to air pollutants promoting chemical protein modification before inhalation and deposition of the proteins in the respiratory tract. Especially summer smog conditions with high O_3_ and NO_2_ concentrations have been shown to efficiently nitrate and cross-link proteins within hours to days (21–24).

Changes of the protein structure and other properties due to nitration and oligomerization can alter the allergenic and inflammatory potential of a protein affecting both, the process of sensitization and the response phase of an allergy (9, 10, 25–31). The development of an IgE-mediated allergy, i.e. sensitization, is a multistep process involving interactions of the innate and adaptive immune systems. The recognition of the allergens by receptors of the airway epithelium, such as the Toll-like receptor 4 (TLR4) and other direct interactions of the allergens with the airway epithelium are the first events after allergen inhalation (32–37). The allergen interactions lead to the release of cytokines, chemokines, and danger signals that initiate the presentation of the allergen to immune cells and the production of allergen-specific IgE, crucial for the allergic response phase. Upon re-exposure to the allergen, cross-linking of IgE antibodies that are surface-bound to effector cells, in particular mast cells, induces cell degranulation and release of pro-inflammatory mediators triggering allergic symptoms (5, 38, 39).

In this study, we investigated the TLR4 activation of the major birch pollen allergen Bet v 1 and the major grass pollen allergen Phl p 5, both being key airborne allergens in Central Europe, before and after chemical modification with ONOO^–^. The proteins were exposed to different amounts of ONOO^–^ in an aqueous phase, and the modifications (tyrosine nitration, oligomerization) were analyzed by liquid chromatography (RP-HPLC, C18) and SDS-PAGE. TLR4 activation and cell viability were determined simultaneously in a stable reporter cell line with bioluminescence detection. Inhibition experiments with the TLR4 antagonist TAK-242 were performed in THP-1-Lucia^TM^ NF-κB cells.

## 2. Materials and methods

### 2.1. Protein and serum samples

Recombinant pollen allergens from birch (*Betula pendula*) and timothy grass (*Phleum pratense*) pollen allergens Bet v 1.0101 and Phl p 5.0101, referred to as Bet v 1 and Phl p 5 hereafter, were obtained from Biomay AG (Vienna, Austria). Ovalbumin (OVA) was purchased from InvivoGen (Toulouse, France) and was treated the same way as the allergens to serve as a negative control in the cell culture experiments described below. Protein stock solutions (1 mg mL^−1^) for chemical modification were prepared with pure water as described in Backes et al. (24).

### 2.2. Protein modification with peroxynitrite

Ammonium bicarbonate (≥98%, Ph. Eur., BP, Carl Roth, Karlsruhe, Germany) was dissolved in pure water to yield a final buffer concentration of 2 M, and the pH was adjusted to 7.8 by the addition of 1 M hydrochloric acid (37% stock solution, Merck Millipore, Darmstadt, Germany). For each reaction, 300 or 500μL of protein solution was transferred into a brown reaction tube (Eppendorf, Hamburg, Germany), and 7.7 or 12.8μL ammonium bicarbonate buffer (2 M) was added to yield a final buffer concentration of 50 mM. After being thawed on ice, sodium peroxynitrite (160–200 mM, Merck Millipore) was added to the protein solutions. To yield molar ratios of ONOO^–^ over tyrosine residues (ONOO^–^/Tyr) of 1/1, 3/1, or 5/1, 0.6, 1.8, or 3μL ONOO^–^ were added to 300μL samples of Bet v 1 and Phl p 5, 4.9μL ONOO^–^ to 300μL samples of OVA (5/1), and 1, 3, or 5μL ONOO^–^ to 500μL samples of Bet v 1 and Phl p 5. The reaction was performed on ice for 110 min. Afterwards, the sample was pipetted into a 10 kDa centrifugal filter (Amicon®, Merck Millipore) and centrifuged at 14 000×g for 2 min (5427 R, Eppendorf). The sample was washed five times with 200μL PBS and centrifugation at 14 000×g for 2 min. For sample recovery, the filter was turned upside down, transferred into a clean microcentrifuge tube, and centrifuged at 1 000×g for 2 min. To recover possible sample residues, the filter was washed with 200μL pure water and centrifuged upside down at 1 000×g for 2 min into the concentrated protein sample. Two to six independent protein samples were prepared for the different ONOO^–^/Tyr ratios. For mock controls of the allergens (termed mock-treated Bet v 1 and mock-treated Phl p 5 hereafter), protein solutions were treated with all buffers but without ONOO^–^, and were purified as described above.

### 2.3. HPLC-DAD analysis

The total tyrosine nitration degree (ND) of the modified samples and controls was determined by HPLC-DAD analysis as described in Selzle et al. (40). Briefly, an HPLC-DAD system (Agilent Technologies 1260 Infinity series, Waldbronn, Germany) equipped with a monomerically bound C18 column (Vydac 238TP, 250mm×2.1mm i.d., 5μm, Hichrom, Berkshire, UK) was used for chromatographic separation. Gradient elution was performed at a flow rate of 0.2 mL min^−1^ with 0.1% (v/v) trifluoroacetic acid (VWR International GmbH, Darmstadt, Germany) in water and acetonitrile (Carl Roth), and absorbance was measured at wavelengths of 220 nm, 280 nm, and 357 nm. The sample injection volume was 10μL, and each chromatographic run was performed in triplicates or duplicates. For system control and data analysis, ChemStation Software was used (Rev. C.01.07, Agilent). The ND is defined as the concentration of nitrotyrosine divided by the sum of the concentrations of nitrotyrosine and tyrosine (40). The protein concentrations of the samples were determined in parallel within the same chromatographic runs using the LC-220 method as described in Reinmuth-Selzle et al. (41).

### 2.4. SDS-PAGE and silver stain

Protein oligomerization was visualized and quantified by silver-stained SDS-PAGE. Protein samples were mixed with an equivalent volume of 2× Laemmli buffer, containing 65.8 mM Tris-HCl (pH 6.8, Carl Roth), 26.3% glycerol (v/v, Carl Roth), 2.1% SDS (Carl Roth) and 0.01% bromophenol blue (Sigma-Aldrich), and heated at 95 ${}^{\circ }$C for 5 min. The samples (75 ng Bet v 1 and 50 ng Phl p 5) were loaded onto a Mini-PROTEAN® TGX™ Precast Protein Gel (4–20%, Bio-Rad, Munich, Germany) together with 60 ng Color Prestained Protein Standard, Broad Range (11–245 kDa or 10–250 kDa, New-England Biolabs, Frankfurt, Germany). Electrophoresis running conditions were constant voltage of 200 V for 40 min. Following electrophoresis, the gels were stained with the Pierce Silver Stain Kit (Thermo Fisher Scientific) following the manufacturer’s protocol. For image acquisition and quantification of protein monomers, dimers, and oligomers, a ChemiDoc system (Bio-Rad) with Image Lab software 6.1 (Bio-Rad) was used. Analysis by SDS-PAGE was performed for two to three independent prepared protein samples.

### 2.5. Endotoxin quantification

The amount of endotoxin in native and ONOO^–^-modified Bet v 1 and Phl p 5 samples was quantified after dilution of the samples to a concentration of 1μg mL^−1^ with endotoxin-free water using the Pierce™ LAL Chromogenic Endotoxin Quantitation Kit (Thermo Fisher Scientific) according to the manufacturer’s protocol. All samples showed less than 0.25 EU per μg of protein.

### 2.6. HeLa TLR4 dual-luciferase reporter cells

For simultaneous determination of TLR4 activity and viability, the well-established HeLa TLR4 dual-luciferase reporter cell line was used (31). In this cell line, Renilla luciferase expressed under the control of an IL-8 promotor serves as a measure for TLR4 activity, whereas a consecutive expressed Firefly luciferase serves as a surrogate marker for cell viability. Cells were grown in Dulbecco’s Modified Eagle’s Medium (DMEM, Thermo Fisher Scientific) containing 25 mM D-glucose, and 1 mM sodium pyruvate supplemented with 10% heat-inactivated fetal calf serum (FCS, Lot #0973F, Biochrom, Berlin, Germany), 1% penicillin/streptomycin (Thermo Fisher Scientific), and 140μg mL^−1^ hygromycin B (InvivoGen) in a humidified atmosphere of 5% CO_2_ at 37 ${}^{\circ }$C. For each experiment, 20 000 HeLa TLR4 dual reporter cells per well were seeded in 100μL complete DMEM in a flat bottom 96-well plate (Greiner, Frickenhausen, Germany). On the next day, the cells were treated with native or modified allergen solutions at a final concentration of 30μg mL^−1^. The samples were diluted with the culture medium to add equal volumes to the cells. Medium, mock-treated allergens, and OVA served as negative controls, and LPS from *E. coli* (LPS–EB, 25 ng mL^−1^, InvivoGen) as a positive control. After 7 h of incubation, cells were washed with 200μL of warm Dulbecco’s PBS containing calcium and magnesium (Thermo Fisher Scientific), followed by lysing the cells using 1× passive lysis buffer (a component of the Dual-Luciferase® Reporter Assay System, Promega, Mannheim, Germany) and freezing at $-80{\;}^{\circ }$C overnight. The Dual-Luciferase® Reporter Assay for analysis of both Renilla and Firefly luciferase reporter activities was performed following the manufacturer’s protocol (Promega). The luminescence signals were measured in a Synergy Neo plate reader (Biotek, Bad Friedrichshall, Germany). To calculate the normalized TLR4 activity, the TLR4-driven Renilla luciferase (TLR4) signal was divided by the Firefly luciferase signal, a surrogate marker for cell viability. The resulting values were normalized to the value of the LPS-treated cells, which was set to 100%. For calculation of viability, the Firefly luciferase (viability) signal was divided by the Firefly luciferase signal of untreated cells and multiplied by 100. Two independent experiments were performed in triplicates. The dose-response relationship of TLR4 activation by Phl p 5 was investigated in additional experiments with final concentrations of 0.25, 0.5, 1, 2.5, 5, 15, 30, and 100μg mL^−1^ of native Phl p 5.

### 2.7. THP-1-Lucia^TM^ NF-κB cells and TLR4 receptor antagonist TAK-242

TLR4 inhibition experiments were performed with THP-1-Lucia^TM^ NF-κB cells (InvivoGen). This immortalized modified THP-1 cell line allows the determination of NF-κB activation by measuring the activity of secreted luciferase. The cells were grown in Roswell Park Memorial Institute (RPMI) 1640 medium (Thermo Fisher Scientific) containing 25 mM D-glucose and 1 mM sodium pyruvate supplemented with 10% heat-inactivated FBS, 100μg mL^−1^ Zeocin™ (InvivoGen), and 1% penicillin/streptomycin in a humidified atmosphere of 5% CO_2_ at 37 ${}^{\circ }$C. For each experiment, 100,000 cells per well were seeded in 50μL medium in a flat-bottom 96-well plate. To inhibit TLR4 signaling, the cells were pre-incubated in duplicates with 500 nM of the TLR4 antagonist TAK-242 (25 mM in dimethyl sulfoxide (DMSO), Merck Millipore, diluted with medium) for 4 h. Medium and medium with DMSO (4.4μg mL^−1^, Sigma-Aldrich) were used as negative controls. LPS-EB ultrapure (25 ng mL^−1^, InvivoGen) served as a positive control. Subsequently, cells were incubated with 30μg mL^−1^ Phl p 5 or control samples for 24 h. Activity of NF-κB was measured by QUANTI-Luc^TM^ reagent (InvivoGen) according to manufacturer’s instructions. Briefly, 10μL of cell culture supernatant was transferred into a white plate (LUMITRAC^TM^, Greiner), and mixed with 50μL of QUANTI-Luc^TM^ reagent. The luminescence was detected in a Synergy Neo plate reader. For each experiment, LPS-treated cells were used as positive control, and the arithmetic mean was set to 100%. This value was used to normalize the measurement results of the Phl p 5 and medium control. Arithmetic mean values and standard deviation were calculated from the normalized values of three independent experiments performed in triplicates (samples without TAK-242) or in duplicates (samples with TAK-242). Assessment of cell viability was performed using the alamarBlue™ cell viability reagent (Thermo Fisher Scientific) according to the manufacturer’s protocol. Excitation was performed at 560 nm, and emission was measured at 590 nm in a Synergy Neo plate reader. Cells treated with DMSO in medium showed no NF–κB activation or toxic effects.

### 2.8. Statistical analysis

GraphPad Prism version 9.0.1 (GraphPad, San Diego, CA, USA) was used for statistical analysis. Unpaired t-tests were performed to observe differences between the native and ONOO^–^-modified proteins.

## 3. Results

### 3.1. Nitration and oligomerization

The two major allergens Bet v 1 and Phl p 5 were exposed to ONOO^–^ in the aqueous phase. Protein nitration was analyzed by reverse-phase chromatography, and protein oligomerization was analyzed by gel electrophoresis. Protein nitration was observed in the native and modified samples of both proteins, and tyrosine nitration degrees were quantified and are summarized in Table 1. The tyrosine nitration degree (ND) is defined as the concentration of nitrotyrosine divided by the sum of the concentrations of tyrosines and nitrotyrosines (40). The native Bet v 1 and Phl p 5 samples had low average NDs of <0.5%. The modified samples were not corrected for these values. For the modified samples, the NDs increase with increasing molar ratios of ONOO^–^ over tyrosine residues (1/1, 3/1, 5/1) and reach maximum NDs of ∼27% (Bet v 1) and ∼24% (Phl p 5). The observed NDs are in good agreement with previous studies (23, 24).

**Table 1:** Nitration degree and relative fractions of monomers, dimers, and oligomers of native, mock-treated, and modified Bet v 1 and Phl p 5 (arithmetic mean values and standard deviations). The allergens were modified with different molar ratios of ONOO^–^ over tyrosine (1/1, 3/1, 5/1). Nitration degrees were determined by reversed-phase HPLC for two to six independent prepared samples measured in triplicates or duplicates, monomer/dimer/oligomer fractions were determined by SDS-PAGE for two to three independent prepared samples.

Sample	Samples measured in reverse-phase HPLC		Nitration degree (%)	Monomer fraction (%)	Dimer fraction (%)	Oligomer fraction (%)
	in triplicates	in duplicates				
native Bet v 1	2	0	0.4±0.1	100	0	0
mock-treated Bet v 1	3	0	0.4±0.1	100	0	0
1/1 Bet v 1	2	0	23.0±3.3	67±0	30±3	3±3
3/1 Bet v 1	3	1	26.9±2.5	66±8	30±3	4±5
5/1 Bet v 1	3	1	27.5±3.8	76±4	23±4	0.3±0.1
native Phl p 5	1	1	0.3±0.1	100	0	0
mock-treated Phl p 5	2	1	0.4±0.1	100	0	0
1/1 Phl p 5	2	3	17.8±1.9	71±4	25±5	4±3
3/1 Phl p 5	3	3	23.3±3.4	67±4	28±3	5±4
5/1 Phl p 5	3	3	24.3±5.5	71±11	23±4	6±7

Protein dimers and higher oligomers were observed in the modified samples of both allergens (Table 1, Figure S1). The native proteins did not contain dimers or oligomers. Of the ONOO^–^-modified Bet v 1 samples, higher dimer and oligomer fractions were found for the samples modified with a lower molar amount of ONOO^–^ over tyrosine (1/1, 3/1), which are probably mimicking more realistic scenarios in the human body. For Phl p 5, dimer and oligomer fractions after ONOO^–^ modification exhibited similarly high values, and agree well with Backes et al. (24), who used size-exclusion chromatography for the determination of the protein oligomer mass fractions of modified Phl p 5.

Besides nitration, dimerization, and oligomerization, the reaction of proteins with oxidants can also result in oxidative side products and protein degradation. The shifts of the retention times and the broadening of the peak widths in reversed-phase chromatography after ONOO^–^ modification indicate changes in hydrophobicity, denaturation, partial unfolding, and the formation of complex reaction products including aggregates and fragments (Figure S2).

### 3.2. TLR4 activation

**Figure 1:**
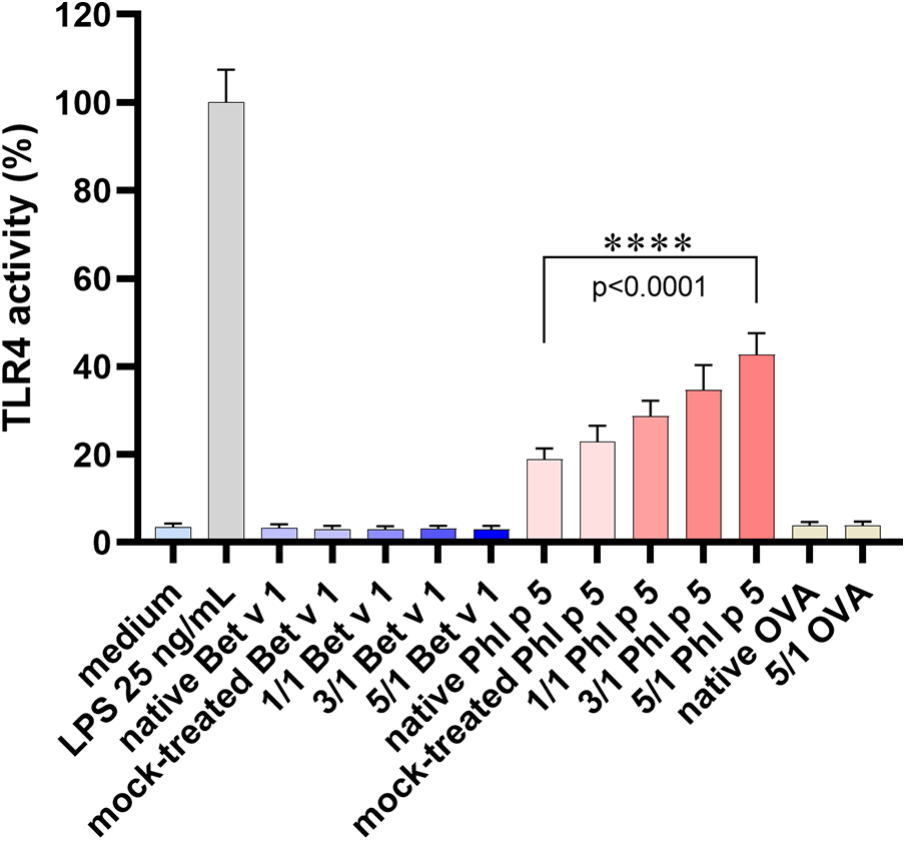
TLR4 activation of native and ONOO^–^-modified allergens. TLR4 activity in HeLa TLR4 dual-luciferase reporter cells determined for Bet v 1, Phl p 5, and OVA after modification with different molar ratios of ONOO^–^ over tyrosine (1/1, 3/1, 5/1) and 7 h of incubation, normalized to LPS. Arithmetic mean values and standard deviations of two independent experiments performed in triplicates.

**Figure 2:**
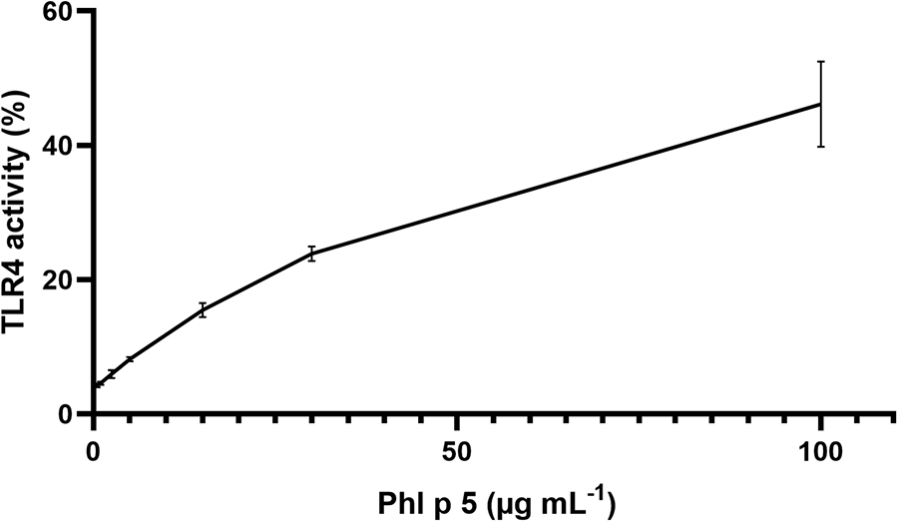
TLR4 activation by different concentrations of native Phl p 5 determined in HeLa dual-luciferase reporter cells and normalized to LPS. Arithmetic mean values and standard deviations for two independent experiments performed in triplicates. The line is to guide the eye.

**Figure 3:**
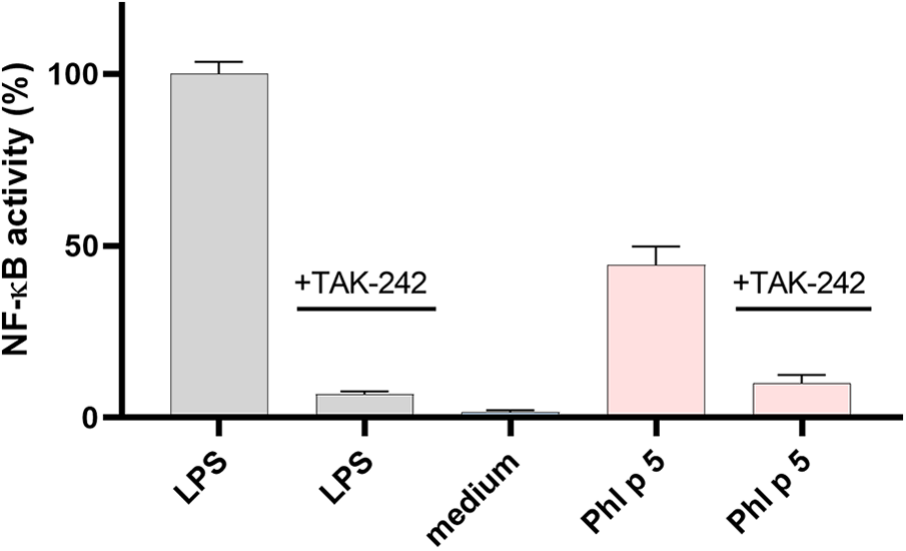
NF-κB activation by native Phl p 5. NF-κB activity in THP-1-Lucia^TM^ NF-κB cells normalized to LPS and inhibition experiments with TLR4 antagonist TAK-242. Arithmetic mean values and standard deviations of three independent experiments performed in triplicates (samples without TAK-242) or in duplicates (samples with TAK-242).

TLR4 activation by the native and modified allergens was determined in a stable HeLa TLR4 dual-luciferase reporter cell line with simultaneous determination of cell viability. Figure 1 shows that native and ONOO^–^-modified Bet v 1 do not activate the TLR4. Native Phl p 5 showed TLR4 activation, and chemical modification with different amounts of ONOO^–^ (1/1, 3/1, 5/1) increased the TLR4 activation of Phl p 5 by factors of ∼1.5 (1/1), ∼1.7 (3/1), and ∼2.1 (5/1) indicating changes in the protein-receptor interaction related to the ONOO^–^ modification. All samples contained less than 0.25 EU endotoxin per μg of protein so that false-positive results due to LPS content can be excluded. The mock-treated Phl p 5 showed increased TLR4 activation by a factor of ∼1.1, indicating that the sample handling induces changes to the protein that affect the interaction with the TLR4. The negative controls of the medium as well as native and ONOO^–^-modified Ovalbumin (OVA) exhibited no substantial TLR4 activation and the applied protein concentrations did not affect the viability of the cells (Figure S3). Additional experiments with different doses of native Phl p 5 showed a dose dependent increase of TLR4 activation (Figure 2) and no effect of the applied protein concentrations on cell viability (Figure S4). The TLR4 activation of Phl p 5 at the highest concentration (∼46% at 100μg mL^−1^) and 5/1 ONOO^–^-modified Phl p 5 (∼43% at 30μg mL^−1^) are similarly high (Figures 1 and 2). Inhibition of the TLR4 by the antagonist TAK-242 in THP-1-Lucia^TM^ NF-κB cells reduced the NF-κB response by 77% (Figure 3) confirming that NF-κB activation induced by Phl p 5 is mediated by the TLR4.

## 4. Discussion

The TLR4 plays a central role in inflammatory processes by recognizing a broad range of pathogen- and damage-associated molecules including bacterial Lipopolysaccharides (LPS) resulting in the release of proinflammatory mediators. Numerous studies indicate a role of the TLR4 in the pathogenesis of allergic diseases (32, 35, 42–46). Direct TLR4 activation by allergens has been reported for the house dust mite proteins Der p 2 and Der p 38 (47–49), wheat amylase trypsin inhibitors (ATI, Baker’s asthma) (50) and for the metal nickel (51). We found TLR4 activation for the grass pollen allergen Phl p 5, but not for the birch pollen allergen Bet v 1. Peroxynitrite modification enhanced the TLR4 activation of Phl p 5, which is in agreement with former studies where we showed that ONOO^–^ modification leads to increased TLR4 activation of the TLR4 stimulating proteins ATI, α-synuclein, heat shock protein 60, and high-mobility group box 1 protein (9, 31). Phl p 5 might act as a multivalent TLR4 ligand due to its two-domain structure and might thus efficiently promote TLR4 dimerization and activation. The enhancement of TLR4 activation of the mock-treated Phl p 5 could indicate that folding changes or protein denaturation contribute to better TLR4 interaction as the mock-treated Phl p 5 exhibited a ND similar low as the native Phl p 5 and behaves as a monomer in the non-reducing SDS-PAGE (Table 1, Figure S1). Also the increase of TLR4 activation by Phl p 5 modified with increasing ONOO^–^ over tyrosine ratios could result from enhanced protein degradation associated with the ONOO^–^ concentrations (23, 40, 52). Dimer and oligomers are less expected to play a role in the enhanced TLR4 activation as the dimer and oligomer fractions exhibited similarly high values for the Phl p 5 modified by the three different ONOO^–^ over tyrosine ratios (Table 1). The concomitant increase of TLR4 activation and nitration degree with increasing amounts of applied ONOO^–^ (Figure 1, Table 1), however, suggests that besides degradation and conformational changes, also tyrosine nitration could play a role in the enhanced TLR4 activation of ONOO^–^-modified Phl p 5. As nitrotyrosine is more acidic than tyrosine, the chemical and physiological properties of a protein such as the isoelectric point and binding to receptors and ligands can change upon nitration (25, 27, 30, 31, 53). Further investigations will be required to determine if and how nitrotyrosine contributes to the enhancement of TLR4 activation by Phl p 5.

The results show that the grass pollen allergen Phl p 5 directly activates the TLR4 and that chemical modification by ONOO^–^ enhances the TLR4 activation and thus the inflammatory potential of Phl p 5. The direct TLR4 activation by Phl p 5 might play a role in the sensitization against the grass pollen allergen and become particularly important during oxidative stress and inflammation. If Phl p 5 is chemically modified by ROS/RNS formed during oxidative stress, innate immune responses can be enhanced through positive feedback loops via TLR4 signaling (9, 54). This amplification of innate immune responses may contribute to increased sensitization to the grass pollen allergen. Further studies are required to better understand and identify the early and most important interactions of the modified allergens with the broad spectrum of pattern recognition receptors on epithelial cells that might contribute to sensitization. Moreover, it is also necessary to analyze how the binding of IgE antibodies and allergic responses are modulated by chemical modification of allergens. The understanding of how environmental risk factors like air pollution, either directly or indirectly via oxidative stress, affect the allergenic potential of proteins is crucial for the protection of public health in the Anthropocene, i.e. the present era of globally pervasive anthropogenic influence on planet Earth and, thus, on the entire human environment (5, 55, 56). Moreover, deeper insights into chemical modifications of allergens and related immune responses can also help in the development of treatments for immune therapy.

## Data Availability

The datasets presented in this study can be found in online repositories. The names of the repository/repositories and accession number(s) can be found below: **The datasets for this study are available at Edmond–the Open Access Data Repository of the Max Planck Society, under https://doi.org/10.17617/3.98W1BB** (57).
