# Supplementary material for: Chemical modification by peroxynitrite enhances TLR4 activation of the grass pollen allergen Phl p 5
Source: Front Allergy. 2023 Feb 15;4:1066392. doi: 10.3389/falgy.2023.1066392 (PMC9975604; doi:10.3389/falgy.2023.1066392)
Supplement: Supplementary file 1 [file Datasheet1.pdf]

Supplementary Material

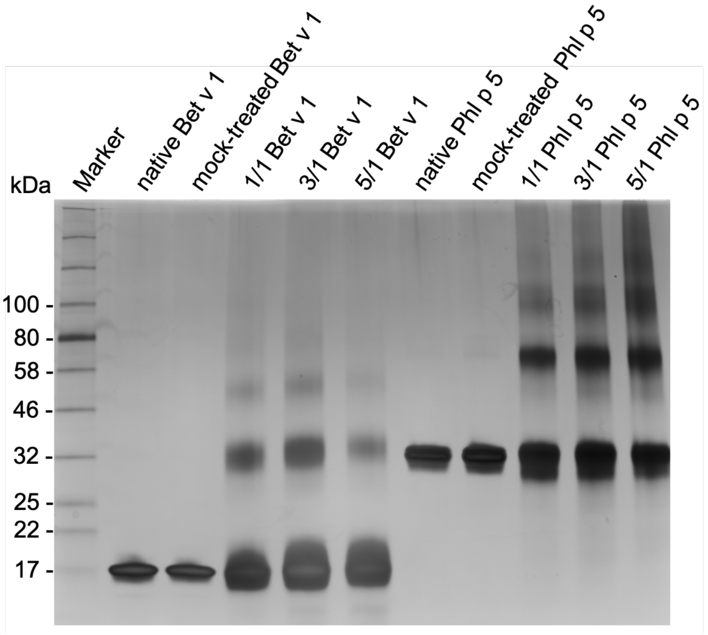

Figure S1. Silver-stained SDS-PAGE gel (non-reducing) of native and modified Bet v 1 and Phl p 5 after reaction with a 1/1, 3/1 or 5/1 molar ratio of $ONOO^{-}$ over tyrosine. Expected molecular masses for Bet v 1: monomer 17 kDa, dimer 34 kDa, trimer 51 kDa, tetramer 68 kDa; and for Phl p 5: monomer 29 kDa, dimer 57 kDa, trimer 86 kDa, and tetramer 114 kDa.

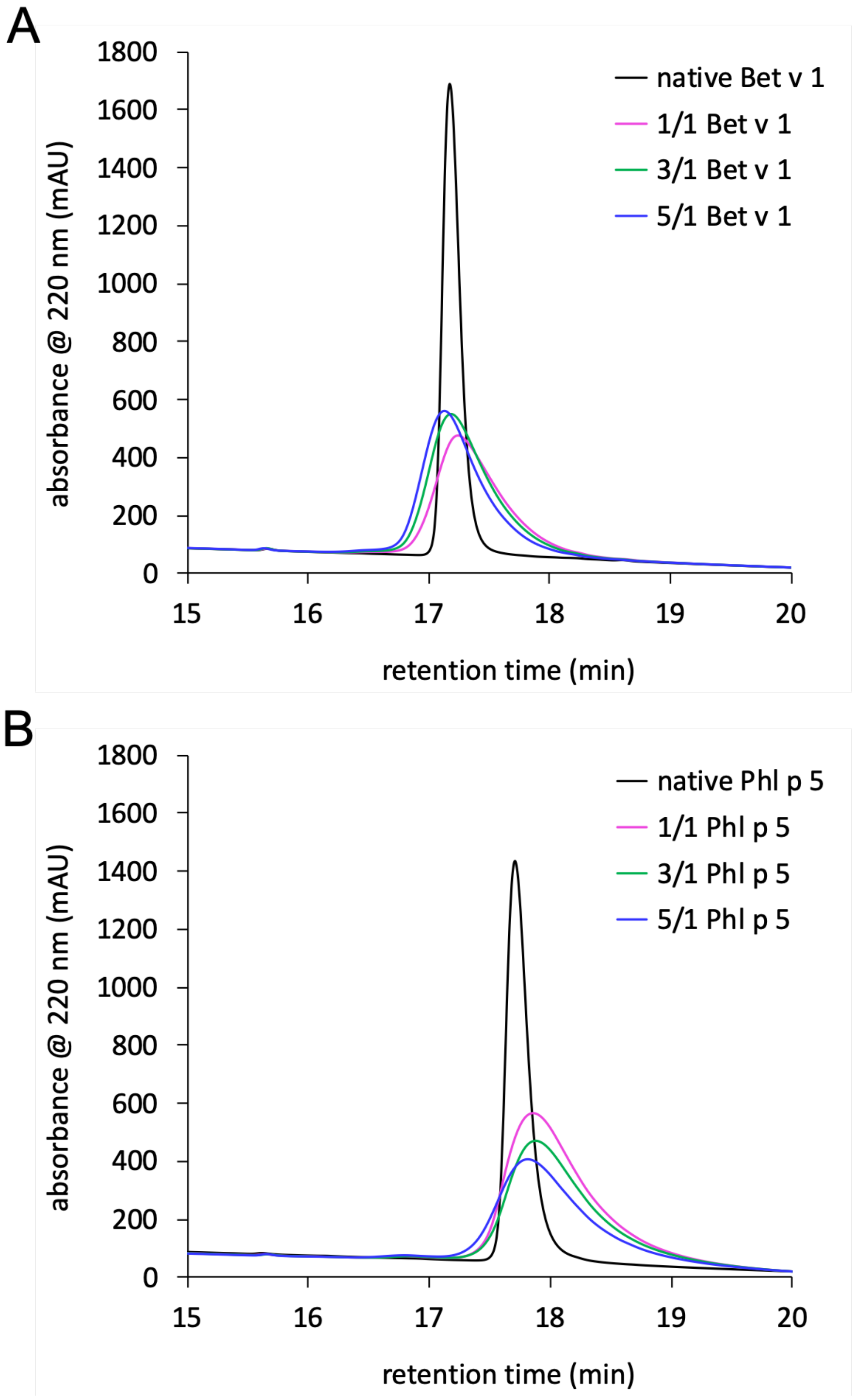

Figure S2. Exemplary chromatograms of reverse-phase HPLC of Bet v 1 (**A**) and Phl p 5 (**B**) before and after reaction with a 1/1, 3/1 or 5/1 molar ratio of $ONOO^-$ over tyrosine.

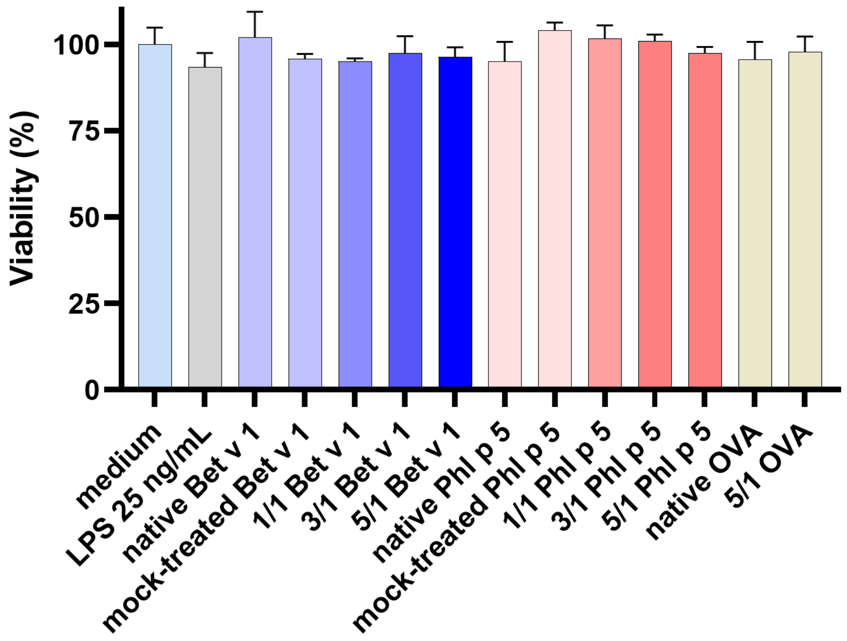

Figure S3. Viability of HeLa TLR4 dual-luciferase reporter cells treated with native and $ONOO^{-}$-modified proteins. Cell viability was assessed using the firefly luciferase luminescence normalized to medium-treated cells. Arithmetic mean values and standard deviations of two independent experiments performed in triplicates.

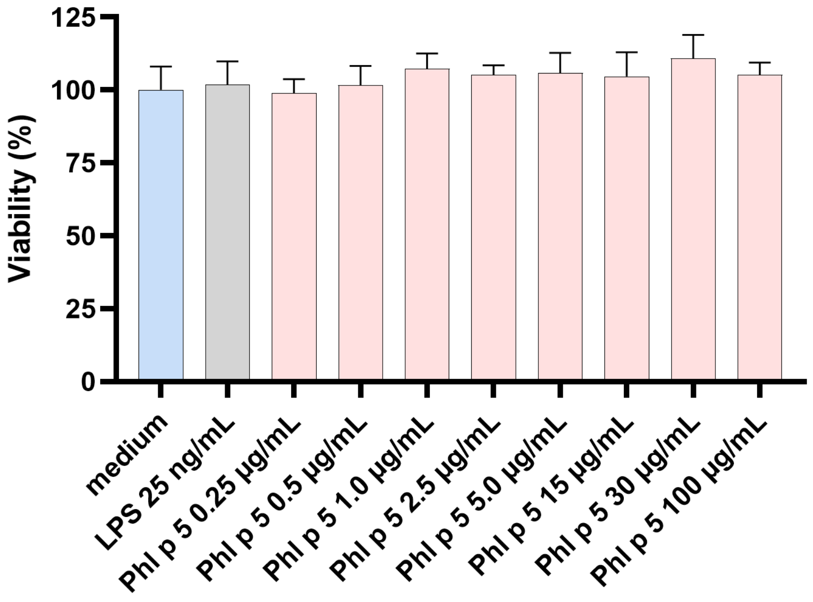

Figure S4. Viability of HeLa TLR4 dual-luciferase reporter cells treated with different concentrations of native Phl p 5. Cell viability was assessed using the firefly luciferase luminescence normalized to medium-treated cells. Arithmetic mean values and standard deviations of two independent experiments performed in triplicates.
